# *OsAlR3* regulates aluminum tolerance through promoting the secretion of organic acids and the expression of antioxidant genes in rice

**DOI:** 10.1186/s12870-024-05298-9

**Published:** 2024-06-28

**Authors:** Chang Su, Jingbo Wang, Jing Feng, Sixu Jiang, Fuyuan Man, Linlin Jiang, Minghui Zhao

**Affiliations:** Rice Research Institute, Collaborative Innovation Center for Genetic Improvement and High Quality and Efficiency Production of Northeast Japonica Rice in China, Shenyang Agricultural University, Shenyang, 110866 China

**Keywords:** *OsAlR3*, Al tolerance, Metabolome, Transcriptome, Organic acids, ROS

## Abstract

In acidic soils, aluminum (Al) toxicity inhibits the growth and development of plant roots and affects nutrient and water absorption, leading to reduced yield and quality. Therefore, it is crucial to investigate and identify candidate genes for Al tolerance and elucidate their physiological and molecular mechanisms under Al stress. In this study, we identified a new gene *OsAlR3* regulating Al tolerance, and analyzed its mechanism from physiological, transcriptional and metabolic levels. Compared with the WT, malondialdehyde (MDA) and hydrogen peroxide (H_2_O_2_) content were significantly increased, superoxide dismutase (SOD) activity and citric acid (CA) content were significantly decreased in the *osalr3* mutant lines when exposed to Al stress. Under Al stress, the *osalr3* exhibited decreased expression of antioxidant-related genes and lower organic acid content compared with WT. Integrated transcriptome and metabolome analysis showed the phenylpropanoid biosynthetic pathway plays an important role in *OsAlR3*-mediated Al tolerance. Exogenous CA and oxalic acid (OA) could increase total root length and enhance the antioxidant capacity in the mutant lines under Al stress. Conclusively, we found a new gene *OsAlR3* that positively regulates Al tolerance by promoting the chelation of Al ions through the secretion of organic acids, and increasing the expression of antioxidant genes.

**Supplementary Information:**

The online version contains supplementary material available at 10.1186/s12870-024-05298-9.

## Introduction

Presently, approximately 50% of the world’s soils (excluding the poles) are acidic, resulting in a significant inhibition of crop productivity [1, 2]. Aluminum (Al), the most abundant metal element in the Earth’s crust, is typically found as an insoluble silicate or alumina compound and is non-toxic to plants growing in normal soil [3]. However, soluble Al^3+^ affects the apical meristematic zone of plants in acidic soils (pH < 5.5), inhibiting root elongation and increasing the plant’s susceptibility to environmental stresses, such as water stress and mineral deficiencies, ultimately inhibiting aboveground growth and leading to decreased yield and quality [4–6]. Due to the large-scale emergence of new agricultural planting models, soil management models, and industrial chemical activities, acid rain is frequently occurring, accelerating the process of soil acidification [7]. The decrease in soil pH and the increase in acidified soil area have become new challenges facing food production. Under the action of acid rain, a large amount of elements such as calcium (Ca), phosphorus (P), and magnesium (Mg) are lost in the soil, while Al gradually precipitates with the action of acid rain, greatly increasing the proportion of harmful Al^3+^ to plants. Rice (*Oryza Sativa L.*) is the world’s second largest food crop after corn. It is also the world’s largest food crop in terms of consumption. Some of the areas where rice is grown have a large amount of acidic soils, and the Al^3+^ in acidic soils will adversely affect the normal growth and development of rice, ultimately affecting the yield and quality of rice. Recently, Al toxicity has become a serious challenge to food security due to increased emission of acidic gases and rapid soil acidification. Therefore, it is crucial to identify Al-tolerance genes in rice and elucidate their physiological and molecular mechanisms under Al stress to provide genetic resources for breeding Al-tolerant rice varieties.

Plants have developed external repulsion and internal tolerance mechanisms against Al stresses [8]. Notably, the external repulsion mechanism can result in the secretion of organic acids by plant roots, which chelate Al^3+^ ions to form nontoxic complexes, thereby reducing Al toxicity to plants [6, 9, 10]. Citric, malic, and oxalic acids are among the most secreted organic acids by plants [11]. Although most graminoids share similar mechanisms of Al tolerance, the mode, type, and quantity of organic acid secretion vary among species. Rice, sorghum, barley, and maize exclusively secrete citrate, whereas Al-tolerant wheat, rye, and oats secrete both citrate and malate [12–16]. Moreover, two distinct patterns of organic acid secretion exist: immediate secretion in barley and wheat upon exposure to Al and delayed secretion in plants such as rice, maize, and sorghum after a period of Al exposure [17]. Enhanced accumulation of citric acid/citrate (CA) improves tolerance to Al, Fe, and alkaline stress [18]. Overexpression of genes encoding organic acid synthesis also leads to increase citrate secretion and improve Al tolerance [19]. Additionally, *TaALMT1* overexpression in barley, wheat, and Arabidopsis increased malate secretion and enhanced Al tolerance [20, 21].

Internal tolerance mechanisms can resist Al toxicity by regulating changes in antioxidant enzyme activity [22, 23]. Various Al-tolerance genes and transcription factors, such as *STOP1*, *ART1*, and *WRKY*, have been identified as key players in the regulation of Al stress [24–26]. Notably, the expression of these genes may be regulated through a complex mechanism involving the co-regulation of repressors and activators of other Al tolerance genes, as well as other control mechanisms of the stress response, such as plant hormones and reactive oxygen species (ROS) [27]. ROS serve as crucial signaling molecules in plant stress responses [28, 29]. Plants experience excessive ROS production within minutes of Al stimulation, leading to oxidative stress and damage to cellular components, including nucleic acids, plasma membranes, and proteins [30, 31]. ROS such as superoxide, hydrogen peroxide, singlet oxygen, and hydroxyl radicals are crucial metabolites produced by plant cells under Al stress [32]. ROS also have positive effects on plant growth, serving as important signaling factors that regulate metabolic and physiological processes and activate antioxidative stress mechanisms to counteract the effects of Al stress [33]. However, ROS homeostasis in plants is determined by the delicate balance between ROS scavenging and generation, and the mechanisms underlying ROS generation and modulation during Al stress remain unclear. ROS are generated in the mitochondria, peroxisomes, and chloroplasts in response to Al stress [34, 35].

In a previous study, we identified a QTL associated with Al resistance on chromosome 1 in rice with a phenotypic contribution of 10.03% using the GWAS [36]. Candidate genes were searched within ± 200 Kb of this QTL locus, and expression level and gene sequence analyses revealed that the relative expression of *OsAlR3* was significantly different under normal conditions and Al stress, and that there were mutations in two bases in the DNA sequence, resulting in changes in the amino acid sequence. The *osalr3* mutant was generated by CRISPR/Cas9, and the analysis of its root length, Al^3+^ content and antioxidant enzyme content revealed significant differences with the WT, which finally determined that the *OsAlR3* was involved in the regulation of Al tolerance in rice seedlings. However, it is important to elucidate the mechanism by which *OsAlR3* induces Al resistance in rice. Therefore, this study aimed to investigate the molecular and physiological mechanisms of *OsAlR3* under Al stress in rice.

## Materials and methods

### Plant materials and stress treatments

Based on previous studies [36, 37], we selected Al-tolerant genotype japonica rice Kunshanzhuozhou and Al-sensitive genotype japonica rice Mangshui from 150 core accessions of Ding as experimental materials, relative root length elongation mean under Al stress were 0.866 and 0.299, respectively. Rice seeds were surface sterilized with 10% (v/v) NaClO for 3 min and soaked in water at 28 °C for 3 d in the dark, then grown hydroponically in the solution of Yoshida in a plant growth chamber (14-h light/10-h dark conditions) with temperatures of 28 °C and 25 °C for the light and dark conditions, respectively. On the 12th day, the rice seedlings were pretreated with Yoshida nutrient solution containing 500 µM of CaCl_2_ (pH = 4) for 24 h. On the 13th day, the rice seedlings were treated with Yoshida nutrient solution containing 100 µM of AlCl_3_ (500 µM of CaCl_2_, pH = 4) for 48 h. Seedlings in the control group were maintained in Yoshida nutrient solution for 14 days.

To investigate the effect of organic acid on Al tolerance, WT plants and *osalr3* mutants in the treatment groups were treated with 100 µM of Al^3+^, 100 µM of Al^3+^+100 µM of citric acid (CA), and 100 µM of Al^3+^+100 µM of oxalic acid (OA) solution (pH = 4.0) for 48 h at the 13th day. Rice seedlings grown in nutrient solution without Al^3+^ (0 µmol/L) were used as the control.

After treatment, 15 WT plants and 15 *osalr3* mutant lines were chosen from each group and their roots were carefully severed and scanned to measure the overall length of the roots using an Expression 1100XL (EU-88, Epson) root scanner.

### Phylogenetic analysis

Sequences of OsAlR3 homologs in other plants were obtained from the NCBI database for Biotechnology Information. Thereafter, sequence alignment was performed using ClustalW in MEGA 11 software. A phylogenetic tree was constructed using the neighbor-joining method. Evolutionary distances were calculated using MEGA 11 through bootstrap analysis (1000 replicates). The tree was annotated using EvolView (https://www.evolgenius.info/evolview/#login).

### Generation of transgenic plants

A pair of sgRNAs was designed using the CRISPR-PLANT website (https://www.genome.arizona.edu/crispr/CRISPRsearch.html). The BsaI sites were incorporated into both the upstream and downstream primers. The sgRNA pair was then annealed to form double-stranded DNA, which was subsequently ligated into the BsaI-digested CRISPR/Cas9 vector pRGEB32 to create a gene-editing vector for *OsAlR3*. To obtain transgenic rice, the constructs were introduced into the mature calli of the Al-tolerant rice genotype Kunshanzhuozhou using the Agrobacterium EHA105-mediated genetic transformation method [38].

### Determination of Al^3+^ content

Briefly, 0.3 g each of the above and below ground parts of WT and *osalr3* mutant lines were digested with 15 mL of HNO_3_ in a graphite ablator (SH420, Manon) at 180 ℃ to yield 1 mL of digest. Thereafter, the digest was fixed to 30 mL and the concentration of Al ions was determined using inductively coupled plasma-atomic emission spectrometry (ICP-MS, Agilent Technologies Inc.).

### Measurement of antioxidant enzyme content in roots

Malondialdehyde (MDA) and H_2_O_2_ contents were determined using the thiobarbituric acid [39] and dimethoate orange [40] methods, respectively. Peroxidase (POD) and superoxide dismutase (SOD) activities were measured using the guaiacol [41], respectively. Superoxide anion (O_2_^−^) content was determined using the hydroxylammonium chloride method [42].

### RNA-Seq and analysis

RNA-seq sequencing and analyses were performed by Novogene Co., Ltd. (Beijing, China). Total RNA was extracted from both WT and *osalr3-1* mutant plants (three biological replicates peer group) using the Plant RNA Prep Kit (QIAGEN, Germany) according the manufacturer’s instructions. Sequencing libraries were prepared and sequenced on a NovaSeq 6000 platform (Illumina, San Diego, CA, United States). Differential expression analysis of two groups was performed using the DESeq2 R package (1.20.0). DESeq2 provide statistical routines for determining differential expression in digital gene expression data using a model based on the negative binomial distribution. The resulting *P*-values were adjusted using the Benjamini and Hochberg’s approach for controlling the false discovery rate. Differentially expressed genes (DEGs) were identified based on significant changes in expression levels (|log_2_fold change| > 1.0, adjusted *p* < 0.05). Gene ontology (GO) functional annotation and Kyoto Encyclopedia of Genes and Genomes (KEGG) pathway enrichment analyses of DEGs were performed using ClusterProfiler software.

### RT-qPCR

Total RNA was extracted from the roots of rice seedlings using a SteadyPure Plant RNA Extraction Kit (Accurate Biology). RNA quality and concentration were assessed using 1% agarose gel electrophoresis and Nanodrop8000 spectrophotometer, respectively. Thereafter, RNA was reverse-transcribed into cDNA using the Evo-M-MLV Reverse Transcription Reagent Premix Kit (Accurate Biology). PCR amplification of target genes was performed by using SYBR Green Pro Taq HS qPCR kit (Accurate Biology) and specific primers. The expression levels of the target genes were calculated using the formula 2^−∆∆CT^ and normalized to that of *OsACTIN1* (internal control). All RT-qPCR reactions were performed three times, and the list of primers used are shown in Supplementary Table 1.

### Metabolome analysis

A comprehensive metabolomic analysis was conducted by Novogene Co. Ltd (Beijing, China) using a SCIEX QTRAP®6500 + mass spectrometer. Briefly, 100 mg of rice root was milled into powder, followed by the addition of 500 µL of 80% methanol solution and centrifugation at 15,000 g for 20 min at 4 °C in an ice bath. The supernatant was diluted with water to obtain a methanol concentration of 53%, and the mixture was centrifuged at 15,000 g for 20 min at 4 °C [43]. For QC samples, equal volumes were mixed for each test sample. The detected metabolites were annotated using the KEGG database (http://www.genome.jp/kegg/pathway.html). Differentially accumulated metabolites (DAMs) were identified using the *t*-test (*p* < 0.05) and variable importance in projection (VIP) ≥ 1.

### Statistical analysis

Data analysis and the drawing of graphs were conducted using relevant software tools such as Excel 2019, IBM SPSS Statistics 22, MEGA 11.0, Origin2018, and Prism. Data are presented as mean ± standard deviation (SD) of data from three replicated trials. Significant differences between groups were determined using one-way analysis of variance (ANOVA), followed Duncan’s multiple range post-hoc test. Statistical significance was set at *p* < 0.05. Graphs sharing the same letter indicate nonsignificant differences among treatments, whereas different letters represent significant differences between treatment groups.

## Results

### Bioinformatic analysis of *OsAlR3*

**Fig. 1:**
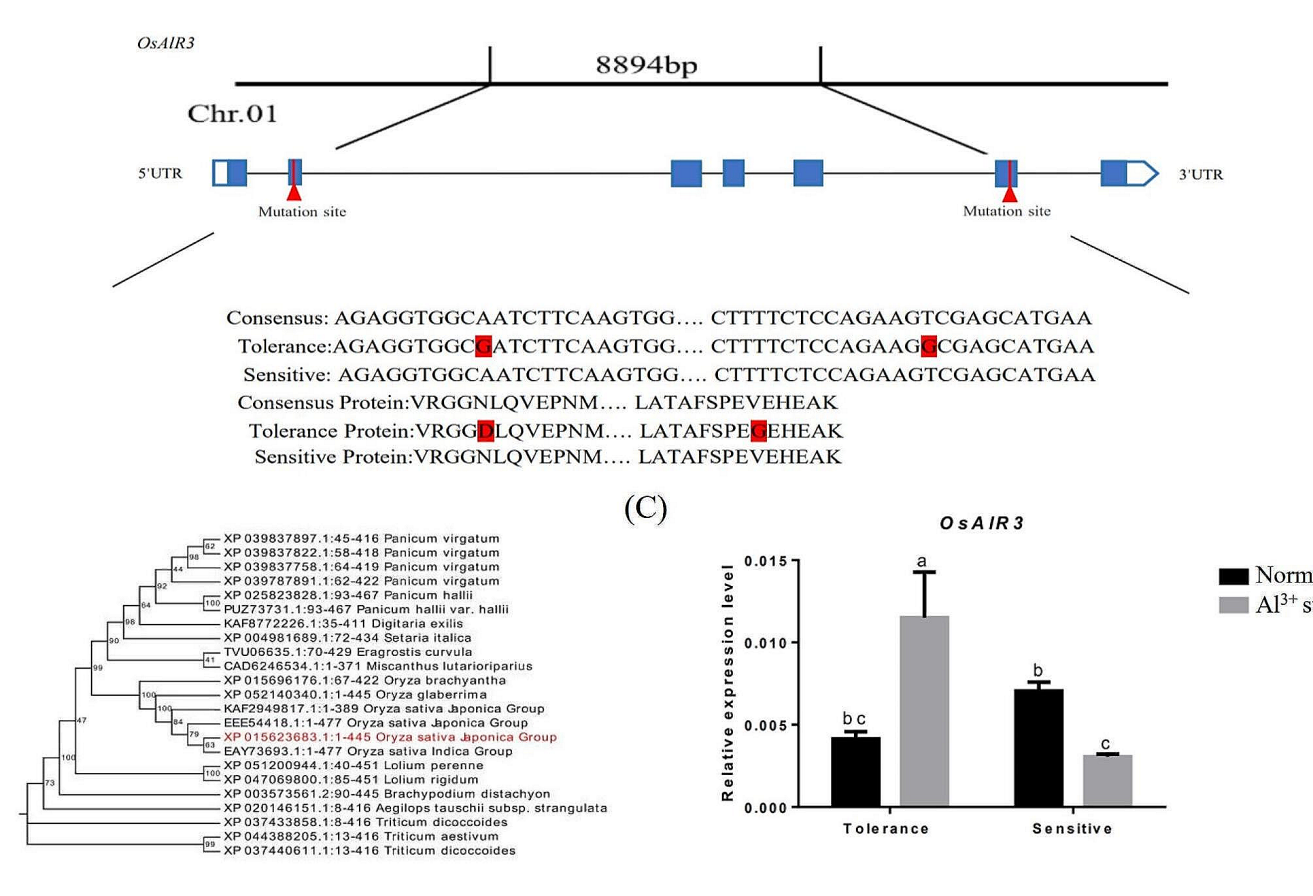
Variation in DNA sequences and relative expression of the *OsAlR3* between the Al-tolerant and -sensitive genotypes of rice. **(A)** Mutations in the *OsAlR3* in both DNA and amino acid sequences of the Al-tolerant and -sensitive genotypes of rice. **(B)** Phylogenetic tree depicting the relationship among OsAlR3 proteins. **(C)** The relative expression of the *OsAlR3* in the Al-tolerant and -sensitive genotypes of rice under normal conditions and Al stress. Different lowercase letters above the bars indicate significant differences (*p* < 0.05 by one-way ANOVA with Duncan’s post-hoc test), all data were presented as the mean value ± SEM (*n* = 3). Rice *OsACTIN1*, was used as an internal control

The *OsAlR3* gene (*LOC_Os01g20110*) is located on chromosome 1 from 11,412,154 to 11,421,047 bp (total length = 8894 bp), with a 115 bp 5’UTR, a 334 bp 3’UTR, and a 1338 bp coding region consisting of seven exons. *OsAlR3* encodes a protein with 445 amino acids. *OsAlR3* belongs to the FOLATE RECEPTOR-LIKE family. Folic acid can promote auxin transport through auxin transporters in roots, and affect root growth and development [44, 45]. A comparison of *OsAlR3* sequence with the Nipponbare genome (reference sequence) showed that the Al-tolerant genotype contained multiple base mutations (Fig. 1A). Notably, the mutation of adenine deoxyribonucleotide (A) to guanine deoxyribonucleotide (G) resulted in the mutation of the encoded L-asparagine (N) to L-aspartic acid (D) and the mutation of thymine deoxyribonucleotide (T) to guanine deoxyribonucleotide (G) resulted in the mutation of valine (V) to glycine (G). No base mutation was observed in the Al-sensitive genotype.

A sequence alignment of the amino acid sequence of OsAlR3 protein (XP 015623683.1) with that of other proteins in the NCBI protein database (https://blast.ncbi.nlm.nih.gov/Blast.cgi?PROGRAM=blastp&PAGE_TYPE=BlastSearch&LINK_LOC=blasthome) identified 22 protein sequences with significant homologies (Fig. 1B). Additionally, a phylogenetic tree constructed using MEGA11 software showed that the closest evolutionary relationships to the OsAlR3 protein were found in Indica, Japonica, and wild rice, with amino acid sequence similarity between the different species and OsAlR3 protein ranging from 73.86 to 100%. Collectively, these data indicates that the protein is relatively conserved in plants and its function may be consistent across different plant species.

To investigate the role of *OsAlR3* in Al tolerance, we examined its expression level in the Al-tolerant and -sensitive rice genotypes. *OsAlR3* expression was significantly upregulated in the Al-tolerant rice genotype, but downregulated in the Al-sensitive genotype under Al stress (Fig. 1C).

### *OsAlR3* positively regulates Al tolerance in rice

**Fig. 2:**
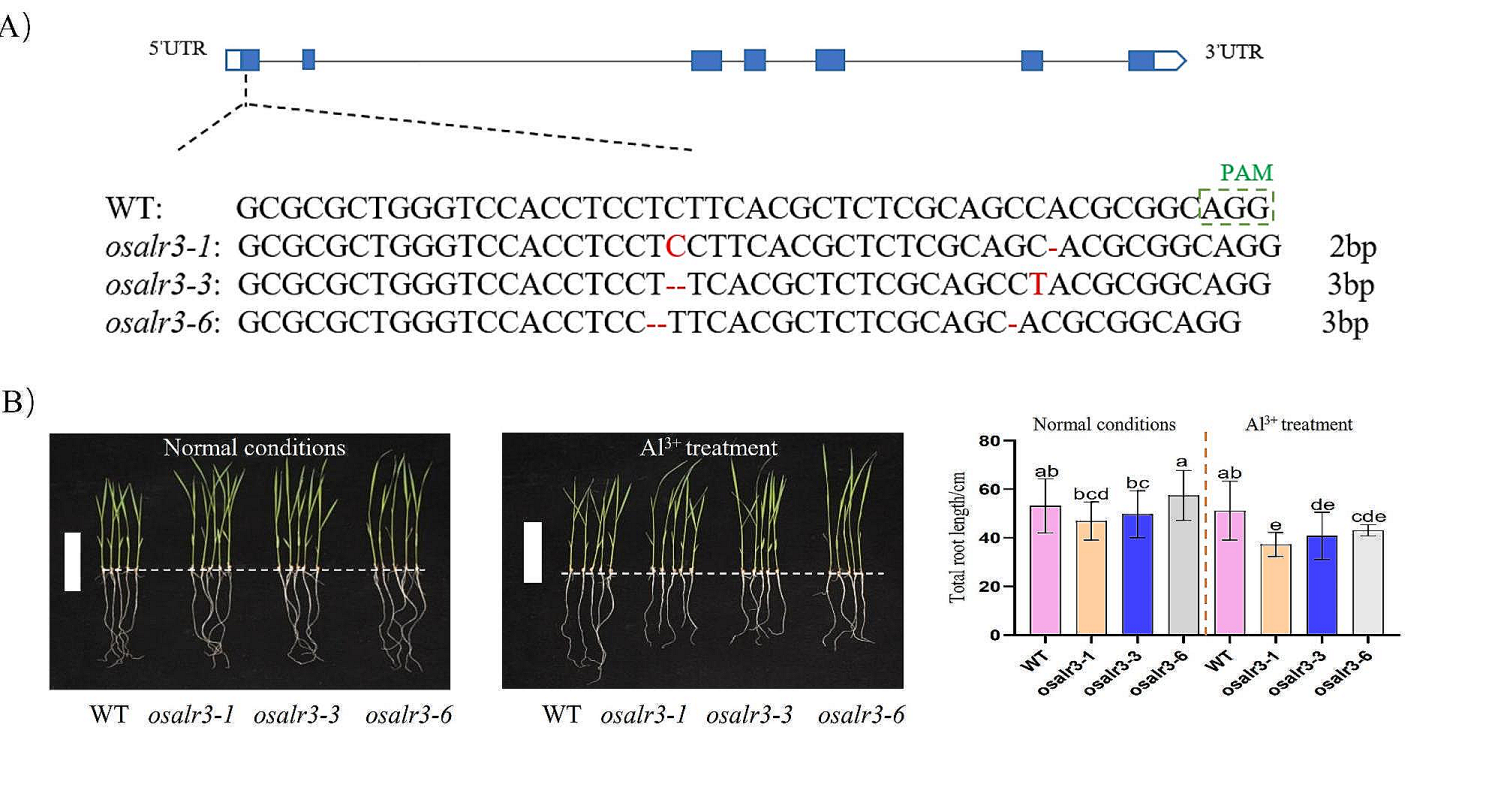
The *osalr3* mutant form and phenotype of mutant lines under Al stress. **(A) **The target sites and CRISPR/Cas9 mutations in the *OsAlR3*. **(B)** The phenotypes of WT and *osalr3* mutant lines under normal conditions and Al stress. Scale bars, 10 cm. Different lowercase letters above the bars indicate significant differences (*p* < 0.05 by one-way ANOVA with Duncan’s post-hoc test), all data were presented as the mean value ± SEM (*n* = 3). The rice seedlings were treated with Yoshida nutrient solution containing 100 µM of AlCl_3_ (500 µM of CaCl_2_, pH = 4) for 48 h

To further confirm the biological function of *OsAlR3*, three *OsAlR3* homozygous mutants were generated using CRISPR/Cas9 technology: *osalr3-1*, *osalr3-3*, and *osalr3-6* (Fig. 2A). In *osalr3-1*, there was an insertion of one base “C” and a deletion of one base “C” in the sgRNA. In *osalr3-3*, there was a deletion of two bases “CT” and an insertion of one base “T” in the sgRNA. In *osalr3-6*, there was a deletion of two bases “TC” and one base “C” in sgRNA. All CRISPR/Cas9-induced mutations resulted in a frameshift in the coding sequence (CDS) of *OsAlR3*, leading to significant truncation or alteration of the OsAlR3 protein in the mutant lines. We examined the total root length of the WT and mutant lines under Al stress (Fig. 2B). The total root length of the mutant lines was significantly shorter under Al stress.

**Fig. 3:**
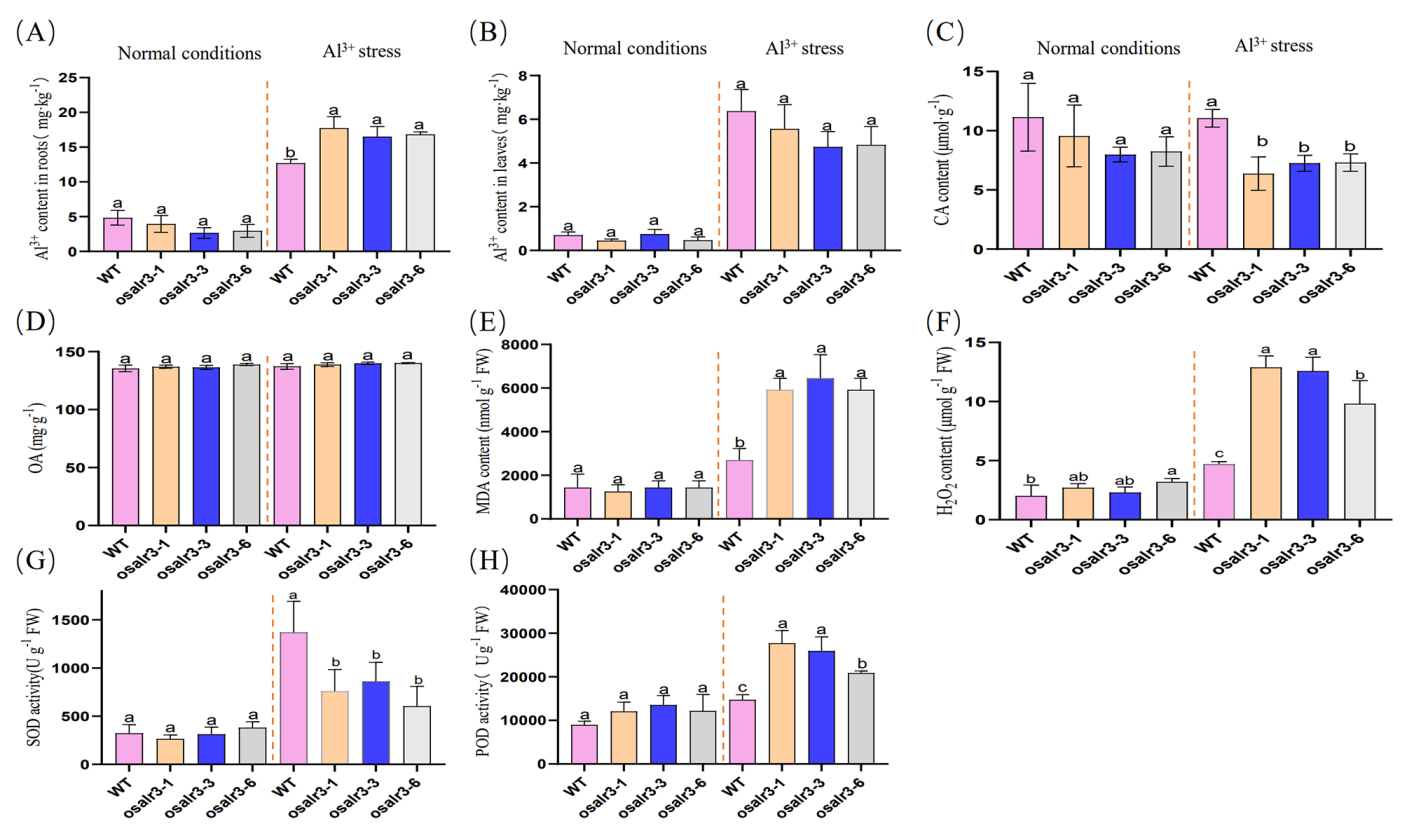
Comparison of Al^3+^ content and physiological indexes between WT and *osalr3* mutant lines under normal conditions and Al stress. **(A)** Al^3+^ content in roots, **(B) **Al^3+^ content in leaves, **(C)** CA content, **(D)** OA content, **(E)** MDA content, **(F)** H_2_O_2_ content, **(G)** SOD activity, **(H)** POD activity. Data represent means ± SEM (*n* = 3). Different letters represent significant difference at *p* < 0.05

Furthermore, we examine the Al^3+^ content of the roots and leaves of the WT and mutant lines (Fig. 3A–B). Under normal conditions, there were no significant differences in Al^3+^ contents in the roots and leaves between the *osalr3* mutant and WT lines. Under Al stress, root Al^3+^ content increased significantly by 39.37%, 29.92%, and 32.68% in the *osalr3-1*, *osalr3-3*, and *osalr3-6* mutants, respectively. Given that the secretion of root organic acids is closely related to Al resistance in rice, we examined the content of CA and OA in the root organic acids of the mutant and WT lines (Fig. 3C–D), and only the content of CA was significantly lower in the mutant lines than in the WT under Al stress. These results suggest that the loss of *OsAlR3* function results in the root system of rice seedlings absorbing more Al ions and secreting less CA under Al stress, aggravating Al toxicity and inhibiting root growth.

To investigate the role of *OsAlR3* on the antioxidant system, we examined MDA and H_2_O_2_ contents and the activities of POD and SOD in the roots. MDA and H_2_O_2_ content were significantly higher in the *osalr3* mutant lines than in WT under Al stress (Fig. 3E–F). The SOD activity of the *osalr3* mutant lines is lower and the POD activity is higher than that of the WT under Al stress. (Fig. 3G–H). Based on the results, it could be speculated that *OsAlR3* may participate in the antioxidant enzyme system to regulate Al tolerance in rice.

### Al stress downregulates the expression of antioxidant-related genes in *osalr3* mutant lines

In total, 12 libraries were sequenced from roots of the WT and *osalr3-1* mutant lines under normal conditions and Al stress. An R^2^ ≥ 0.86 between biological replicates indicated good reproducibility of results and reliability of the experiment (Fig.S1A). Principal component analysis (PCA) indicated that the four samples formed distinct clusters (Fig.S1B). The RNA-Seq data were of high quality, as evidenced by the high percentage of clean reads (Q30 and Q20) and lower percentage of low-quality sequences, error rates, and reads containing poly-N and adaptors (Supplementary Table 2).

Differential expression analysis was performed to identify differentially expressed genes (DEGs) in response to Al stress using the following cutoff: *p* adj. < 0.05, and |log_2_fold change| > 1.0. In total, 297 DEGs (165 upregulated and 132 downregulated) (Fig.S1C; Supplementary Table 3) and 178 DEGs (383 upregulated and 1405 downregulated) (Fig.S1D; Supplementary Table 4) were identified in the *osalr3* vs. WT groups under normal conditions and Al stress. The number of DEGs was significantly higher under Al stress than under normal conditions. Under Al stress and normal conditions, we identified 665 DEGs (360 upregulated and 305 downregulated) in the WT plants (Fig. S1E; Supplementary Table 5) and 927 DEGs (202 upregulated and 725 downregulated) in the *osalr3* lines (Fig.S1F; Supplementary Table 6). Overall, the number of DEGs was significantly higher in the *osalr3* mutant lines than that in the WT plants under normal conditions and Al stress.

**Fig. 4:**
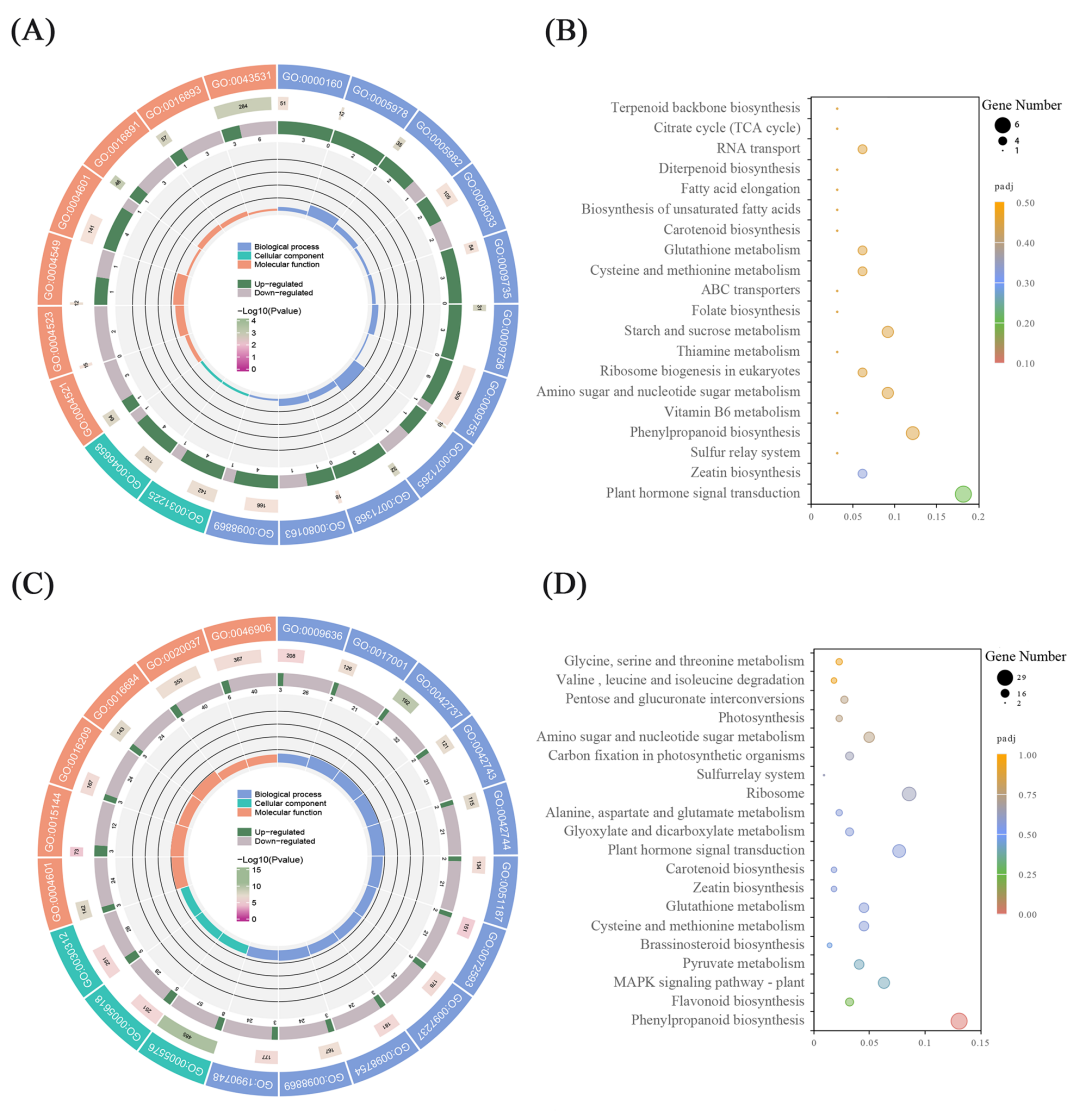
The Gene Ontology (GO) and Kyoto Encyclopedia of Genes and Genomes (KEGG) enrichment Analyses. **(A)** The Gene Ontology (GO) enrichment of DEGs between WT and the *osalr3* lines under normal conditions. **(B)** The KEGG enrichment of DEGs between WT and the *osalr3* lines under normal conditions. **(C) **The Gene Ontology (GO) enrichment of DEGs between WT and the *osalr3* lines under Al stress.** (D) **The KEGG enrichment of DEGs between WT and the *osalr3* lines under Al stress

GO analysis indicated that DEGs under normal conditions were mainly associated with endoribonuclease activity, 5’-phosphomonoesters production (GO:0016111), and ADP binding (GO:0043531) (Fig. 4A), and DEGs were enriched in drug catabolic process (GO:0042737), hydrogen peroxide catabolic process (GO:0042744), peroxidase activity (GO:0004601), and extracellular region (GO:0005576) under Al stress (Fig. 4C). KEGG analysis indicated that most DEGs were enriched in plant hormone signal transduction, zeatin biosynthesis, and phenylpropanoid biosynthesis under normal conditions (Fig. 4B), and significantly enriched in phenylpropanoid and flavonoid biosynthesis under Al stress (Fig. 4D).

GO enrichment analysis revealed that downregulated DEGs in *osalr3* were involved in the peroxidase activity, oxidoreductase activity, acting on peroxide as an acceptor, and hydrogen peroxide catabolic processes. We focused on the stress-related genes. Such as *OSPME28* and *OSPME1*, which encode pectin methyl esterase (PME), *PER1A*, which encodes peroxide reductase, and *OSAPX8*, which encodes ascorbic acid Peroxidase, have decreased expression in the *osalr3* mutant lines.

**Fig. 5:**
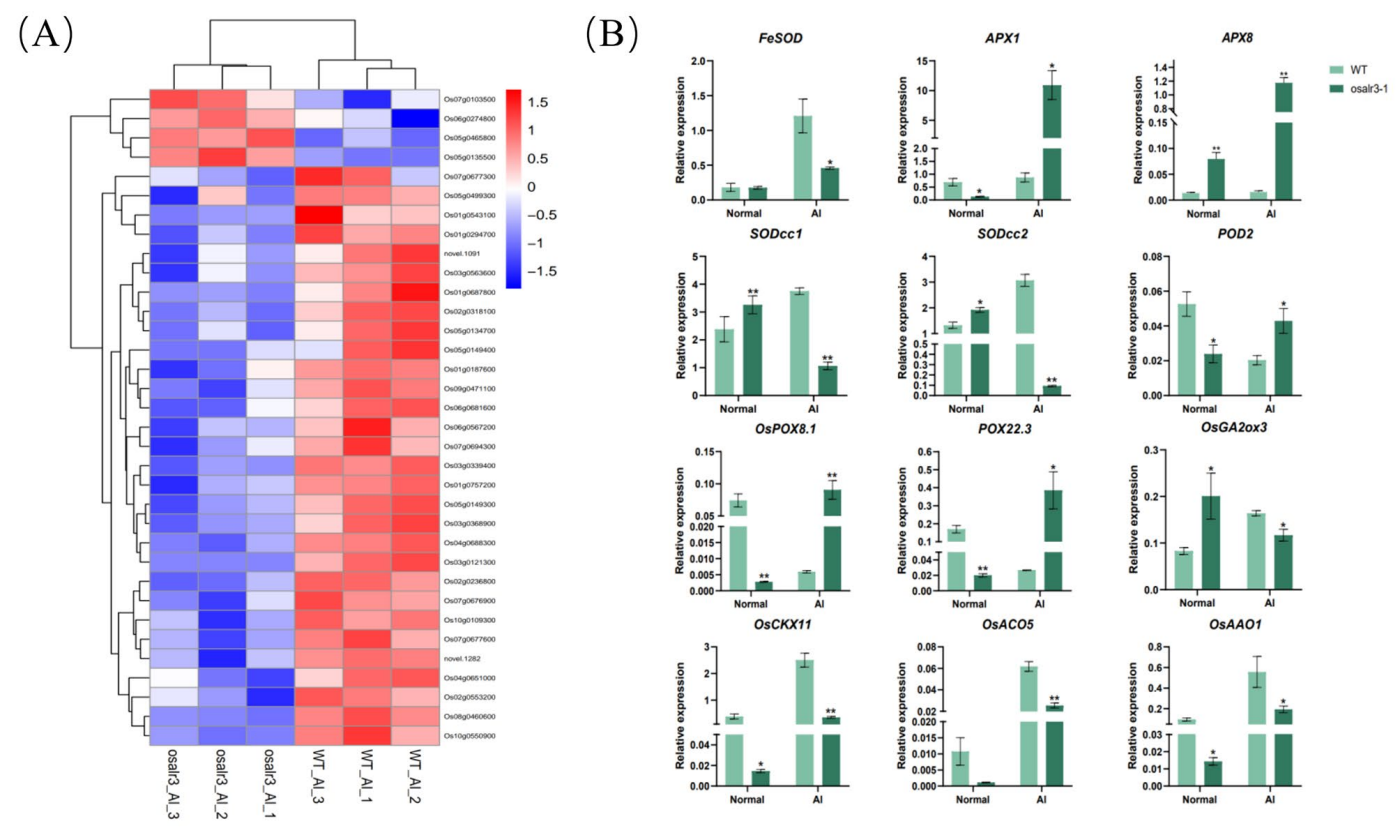
DEGs between WT and *osalr3* lines were involved in the oxidation-reduction process. **(A)** Heatmap showing the enriched genes in terms of oxidation-reduction process. **(B) **Relative expression of genes related to encoded peroxidase and ROS scavenging in WT and *osalr3* lines under normal conditions and Al stress. Data represents means ± SEM (*n* = 3). A two-tailed Student’s t-test was used to compare the difference of data from two groups. Rice *OsACTIN1*, was used as an internal control. *P < 0.05, **P < 0.01

Based on the physiological differences between the WT and *osalr3* lines, we focused on the oxidation-reduction process. 34 were peroxidase coding genes (Supplementary Table 7), of which 30 were significantly downregulated and 4 upregulated under Al stress (Fig. 5A). RT-qPCR was performed to examine the expression of eight ROS-related genes (Fig. 5B). *FeSOD*, *SODcc1*, *SODcc2*, *OsGA2ox3*, *OsCKX11*, *OsACO5* and *OsAAO1* expression levels were significantly lower and *APX1*, *APX8*, *POD2*, *OsPOX8.1*, and *POD22.3* expression levels were significantly higher in the *osalr3* lines than in the WT plants under Al stress. Overall, these results indicate that *OsAlR3* plays a key role in maintaining redox homeostasis.

To validate the results of the RNA-seq, RT-qPCR was performed to examine the expression of five randomly selected DEGs (Fig. S2). The expression trends of the five DEGs were consistent with the transcriptomic data, which indicated that the expression data in transcriptomic analysis were reliable.

### Al stress affects the organic acid profile in *osalr3* mutant lines

A comprehensive class-targeted metabolome analysis was performed to analyze differences in metabolites between WT and *osalr3* lines under normal conditions and Al stress. Hierarchical cluster analysis (HCA) and PCA indicated significant separation between the samples (Fig.S3A-B). PLS-DA was performed to assess the stability of the model (Fig.S3C-F). The original PLS-DA model demonstrated high stability and reliability, as evidenced by the close-to-1 values for both R2 and Q2. The models were ranked and validated to determine their quality. The R2 values obtained in each group were higher than those in Q2, and the intercept of the Q2 regression line with the Y-axis was less than 0, suggesting that these models were of good quality and yielded meaningful results (Fig. S4A–D).

.

In total, 889 metabolites were detected in the four groups (Supplementary Table S8). Differentially accumulated metabolites (DAMs) were identified using a multivariate PLS-DA model, based on variable importance in projection (VIP) > 1.0, fold change (FC) > 1.5 or < 0.667, and *p* < 0.05. In total, 259 DAMs (164 upregulated and 95 downregulated) were identified under Al stress (Supplementary Table 9), and 268 DAMs (202 upregulated and 66 downregulated) were identified under normal conditions (Supplementary Table 10). Additionally, 143 DAMs (114 upregulated and 29 downregulated) were identified in the WT plants (Supplementary Table 11) and 210 DAMs (121 upregulated and 89 downregulated) were identified in the *osalr3* mutant lines under Al stress and normal conditions (Supplementary Table 12). Collectively, these results indicate that *OsAlR3* plays a role in regulating the production of various metabolites.

**Fig. 6:**
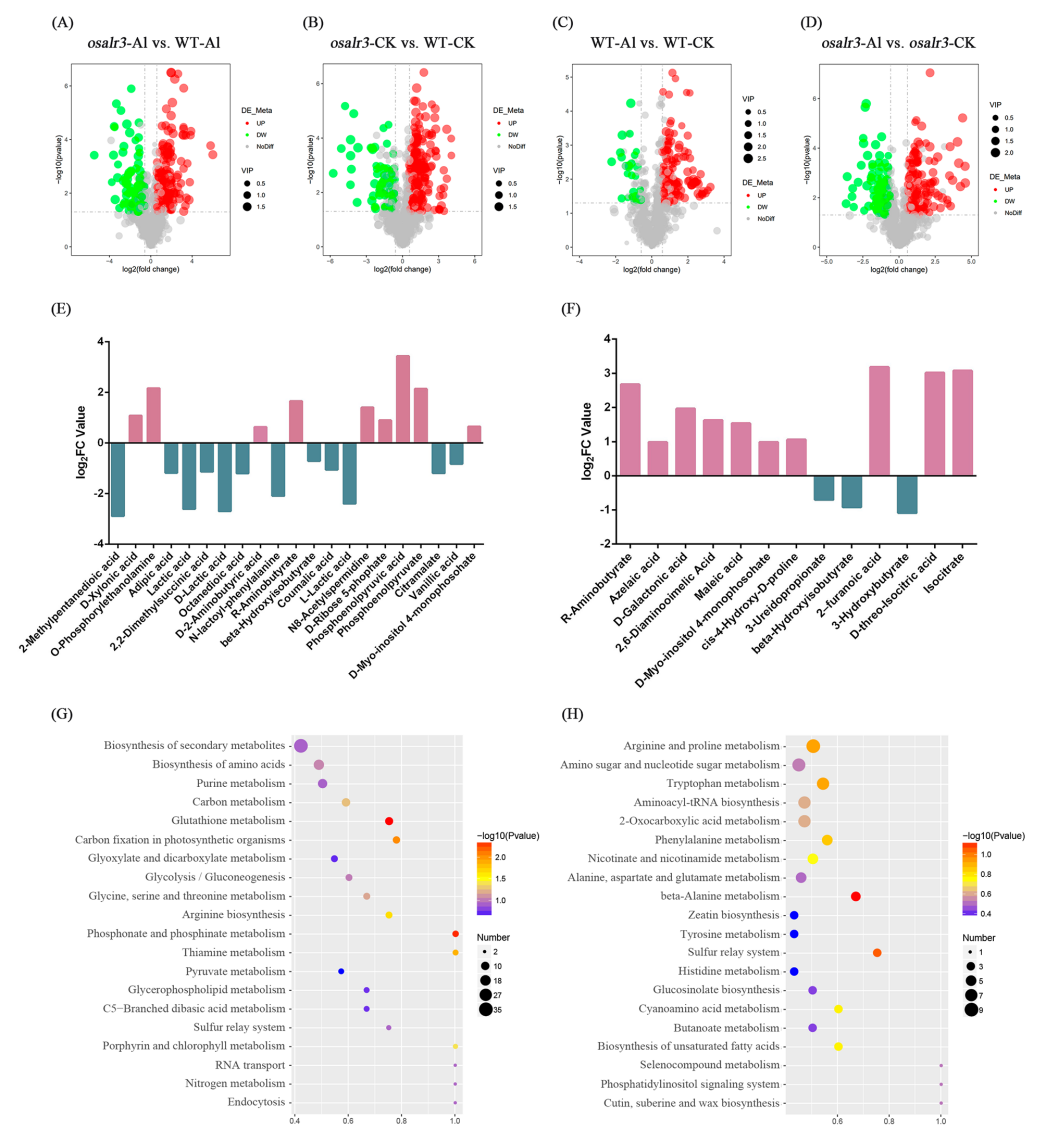
Differentially accumulated metabolites (DAMs) between WT and the *osalr3* lines under normal conditions and Al stress. **(A)** Volcano plots of the metabolites comparing *osalr3* and WT under Al stress. **(B)** Volcano plots of the metabolites comparing *osalr3* and WT under normal conditions. **(C)** Volcano plots of the metabolites comparing WT under Al stress and normal conditions. **(D)** Volcano plots of the metabolites comparing *osalr3* under Al stress and normal conditions. **(E)** Organic acids in the *osalr3*-Al vs. WT-Al group. **(F)** Organic acids in the *osalr3*-N vs. WT-N group. (G) The top 20 KEGG pathways of DAMs under Al stress. **(H)** The top 20 KEGG pathways of DAMs under normal conditions

Further analyses were performed on DAMs in the *osalr3* vs. WT lines. The expression patterns of metabolites were similar among biological replicates, but significantly different between the WT and *osalr3* lines (Fig. 6A–D). Specifically, we identified several organic acids with distinct accumulation patterns in the WT and *osalr3* lines (Fig. 6E–F). Compared with that the WT group, 12 out of 21 organic acids, including lactic acid, coumaric acid, and vanillic acid, were significantly downregulated in the *osalr3* lines under Al stress. 3 out of 13 organic acids (3-ureidopropionate, β-hydroxyisobutyrate and 3-hydroxybutyraye) were significantly downregulated in the *osalr3* vs. WT groups under normal conditions. Overall, these results suggest that *OsAlR3* knockout may affect the organic acid profile of rice roots under Al stress.

KEGG analysis of the DAMs was performed to obtain information on the pathways associated with the metabolites. There were significant changes in glutathione, phosphonate, and phosphinate metabolism in the *osalr3* mutant lines under Al stress (Fig. 6G). Under normal conditions, β-alanine metabolism and the sulfur relay system were highly enriched (Fig. 6H).

### *OsAlR3* modulates phenylpropanoid biosynthesis

Integrated metabolome and transcriptome analysis was performed to investigate the relationship between gene expression and metabolite synthesis. 35 KEGG pathways, including phenylpropanoid biosynthesis (29 genes), pyruvate metabolism (9 genes), and glutathione metabolism (10 genes), were highly enriched in the *osalr3* vs. WT groups under Al stress (Fig. S5A). Similarly, 10 KEGG pathways, including zeatin biosynthesis (2 genes) and phenylpropanoid biosynthesis (4 genes), were highly enriched in the *osalr3* vs. WT groups under normal conditions (Fig. S5B). DEGs associated with the phenylpropanoid biosynthesis pathway showed significant differences (*p* < 0.01) in the *osalr3* vs. WT groups under normal conditions and Al stress. This result suggests that phenylpropanoid biosynthesis plays a crucial role in Al tolerance in rice.

**Fig. 7:**
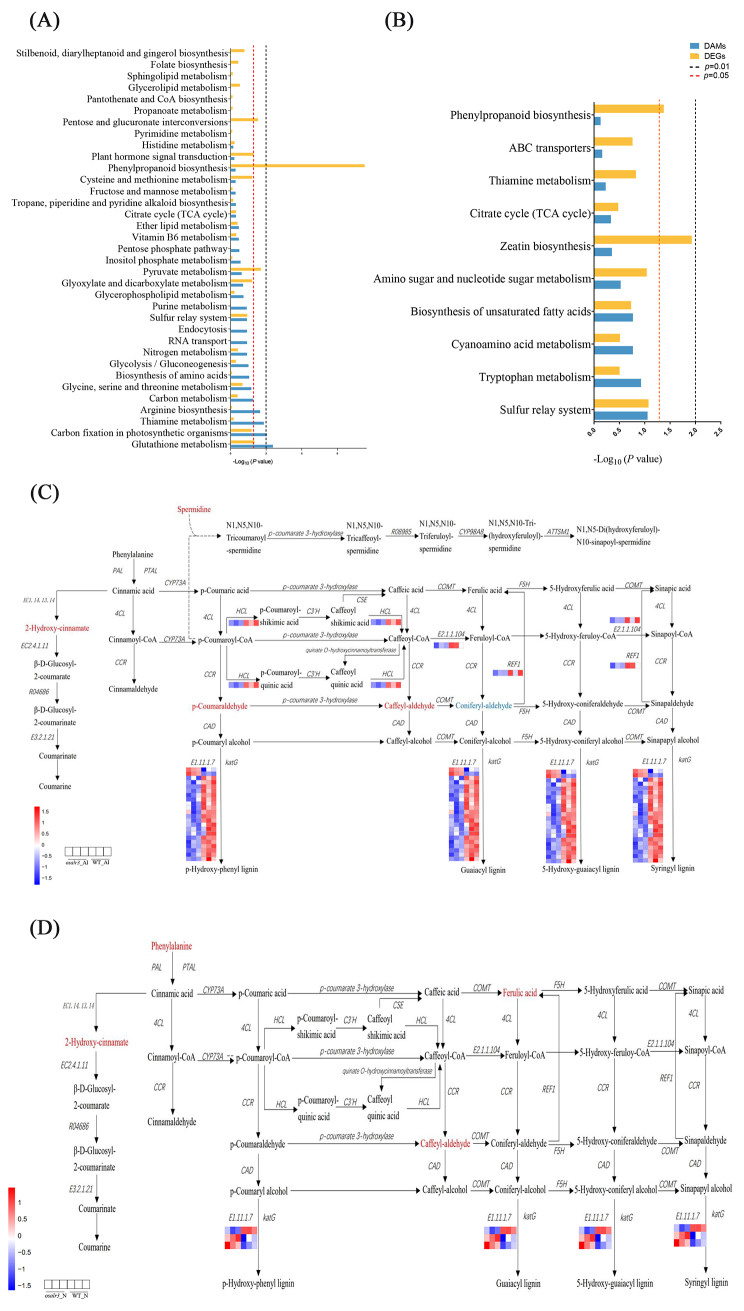
Statistics of KEGG pathways for DAMs and DEGs. **(A)** KEGG pathways enriched in *osalr3*-Al vs. WT-Al. **(B)** KEGG pathways enriched in *osalr3*-N vs. WT-N. **(C)** Different accumulation and expression patterns of metabolites and genes related to the phenylpropanoid biosynthesis pathway in *osalr3*-Al vs. WT-Al. **(D)** Different accumulation and expression patterns of metabolites and genes related to the phenylpropanoid biosynthesis pathway in *osalr3*-N vs. WT-N. The differentially accumulated metabolites are shown in red and blue. The expression levels of genes are shown from red to blue (high to low) in the comparison of WT-Al vs. *osalr3*-Al and WT-N vs. *osalr3*-N. Gene heatmap shows the value of p adj in WT (left panel) and *osalr3* knockout lines (right panel). WT-N, WT under normal conditions. *osalr3*-N, *osalr3* lines under normal conditions. WT-Al, WT under Al stress. o*salr3*-Al, *osalr3* lines under Al stress

To further investigate the role of the phenylpropanoid biosynthesis pathway in Al tolerance, we screened DEGs and DAMs associated with the pathway (Fig. 7A–B). 2-Hydroxycinnamate, spermidine, p-coumaraldehyde, and caffeoyl-aldehyde contents were significantly higher and coniferyl-aldehyde was significantly lower in the *osalr3* lines than in the WT under Al stress (Fig. 7C). Notably, there were significant differences in the expression of 25 genes in the phenylpropanoid biosynthesis pathway between the *osalr3* and WT groups under Al stress. Among the genes regulating E1.11.1.7 (peroxidase), 20 downregulated genes and 2 upregulated genes were in the *osalr3* lines under Al stress. Genes encoding shikimate O-hydroxycinnamyltransferase (HCT; *Os09g0422000*), caffeoyl-CoA O-methyltransferase (E2.1.1.104; *Os09g0481400*), and coniferyl-aldehyde dehydrogenase (REF1; *Os01g0591300*) were significantly downregulated in the *osalr3* group under Al stress. Under normal conditions, phenylalanine, 2-hydroxy-cinnamate, ferulic acid, and caffeoyldehyde were upregulated in the *osalr3* group compared with that in the WT group (Fig. 7D). The expression of *Os01g0294700* increased by 1.38-fold in the *osalr3-*N vs. WT-N group, but decreased by 1.26-fold in the *osalr3*-Al vs. WT-Al group. Similarly, the expression of *Os07g0676900* was increased by 1.88-fold under normal conditions but reduced by 1.84-fold under Al stress. The expression of *Os07g0638300* was reduced by 2.55-fold and 1.64-fold in the *osalr3-*N vs. WT-N and *osalr3*-Al vs. WT-Al groups, respectively. Collectively, these results suggest that *OsAlR3* plays a crucial role in the phenylpropanoid biosynthesis pathway in response to Al stress.

### Exogenous organic acids enhanced the resistance of *osalr3* mutant lines to Al toxicity

**Fig. 8:**
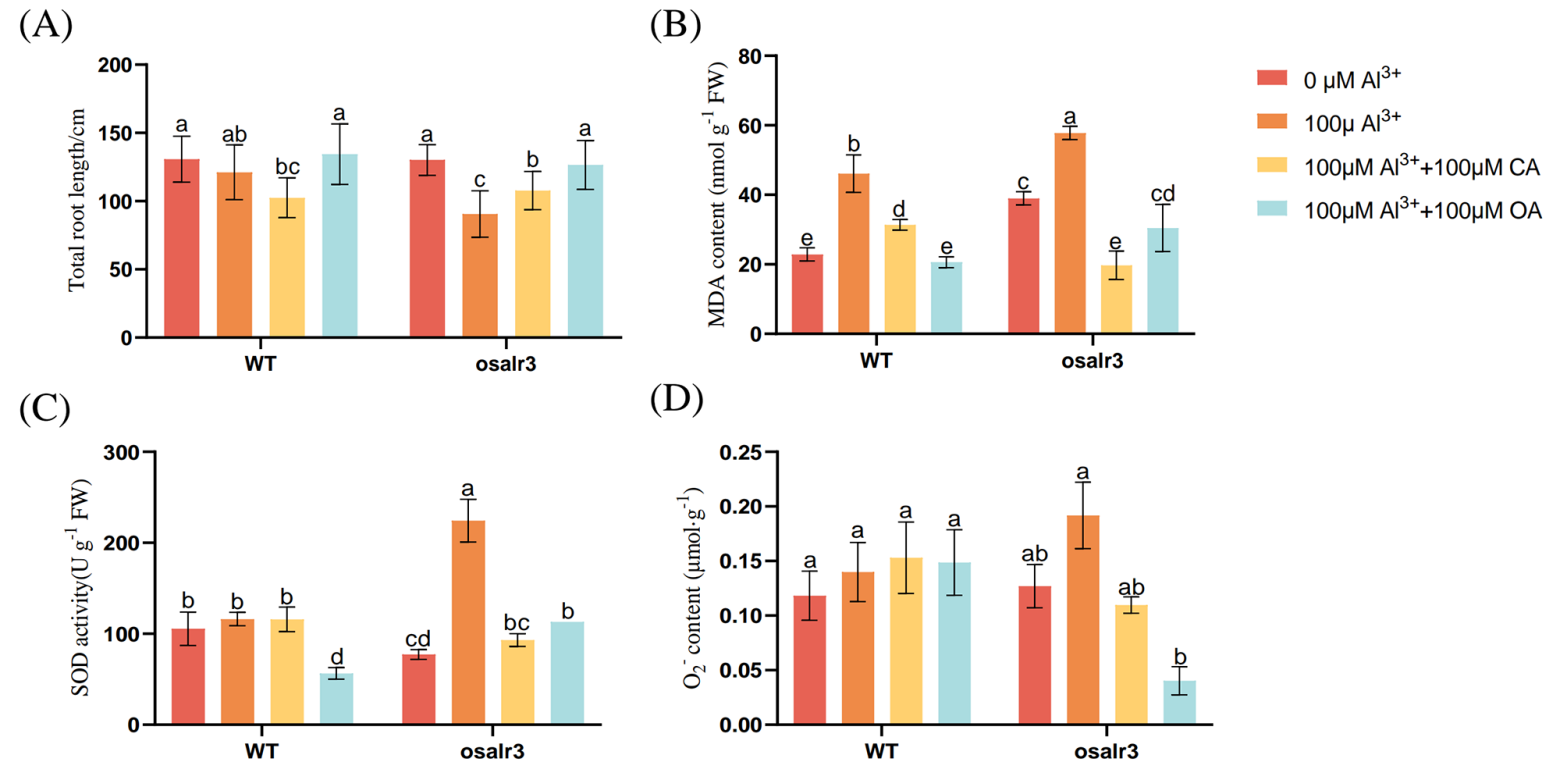
Responses of *osalr3* mutant lines and WT plants to exogenous organic acids under Al stress. **(A) **Total root length, **(B)** MDA content, **(C)** SOD activity, **(D)** O_2_^−^ content. Data represent means ± SEM (*n* = 3). Different letters represent significant difference at *p* < 0.05

Metabolomic analysis showed that there was a significant difference in organic acid profile between WT and *osalr3* mutant lines under Al stress. Therefore, we speculated that *OsAlR3* may play a regulatory role in the secretion of organic acids (Fig. 8). To test this hypothesis, rice seedlings exposed to Al stress were treated with 100 µΜ of CA and OA. Compared with Al stress, exogenous CA and OA increased total root length, decreased the content of MDA, O_2_^−^and SOD activity in the *osalr3* mutant lines, but had less effect on the WT. Overall, these results indicate that exogenous organic acids may attenuate the toxic effects of Al in *osalr3* mutant lines.

## Discussion

Several crops are susceptible to Al toxicity in acidic soils, severely limiting their development and yield. Al is a reactive element that exerts toxicity through various mechanisms, including interactions with the cell wall, exoplasm, and ectoplasm. Al tolerance in rice is a complex trait controlled by multiple genes, and the mechanism of Al toxicity in rice remains unknown [46]. Therefore, it is crucial to screen and identify candidate genes associated with Al tolerance to elucidate the underlying mechanisms of Al toxicity in rice. In the previous study, we found that the *OsAlR3* gene is a potential regulator of Al tolerance in rice seedlings. Folic acid enhances auxin transport through auxin transporters, impacting root growth and development. The Auxin 1/LIKE AUX1 family member *OsAUX1* mitigates Al-induced oxidative damage by suppressing auxin transport [47]. Furthermore, Al stress was found to be associated with reduced auxin accumulation in root tips, a process regulated by *ZmPGP1*, ultimately hindering root growth [48]. The application of exogenous Naphthalen-1-Yl-Acetic Acid (NAA) in barley amplified the inhibition of Al-induced root growth by auxin. Additionally, Al treatment triggered the expression of auxin-responsive genes in root tips [49]. We have searched for *Os08g0320800* in rice, but the function of this gene is not clear. The homologous gene *AT4G37920* was also found in Arabidopsis thaliana. *AT4G37920* is involved in plastid RNA processing and is essential for chloroplast development and biogenesis [50, 51]. In the present study, morphological, physiological, metabolomic, and transcriptomic analyses were performed in WT and *osalr3* mutant lines under normal conditions and Al stress to elucidate the mechanism of *OsAlR3* in Al tolerance.

The most recognizable characteristic of Al toxicity is the inhibition of root elongation [52]. Additionally, Al toxicity can cause root hair hypoplasia, root tip swelling, leaf necrosis, and reduce yield [53]. Morphological analysis showed that the *osalr3* mutant lines had significantly shorter root length than the WT plants under Al stress (Fig. 2). Moreover, *OsAlR3* knockout increased the sensitivity of the mutants to Al stress, suggesting that *OsAlR3* plays a positive role in Al tolerance in rice seedlings. Similarly, *miR393* overexpression substantially alleviated Al-induced root growth inhibition in barley, whereas *MIM393* knockdown enhanced the sensitivity of the roots to Al stress [49].

Under normal conditions, ROS production and elimination in plants are relatively stable; however, various abiotic stresses, such as salt, high temperature, and heavy metal stress, induce excessive ROS generation, resulting in oxidative damage and destruction of cell metabolism [54, 55]. Under Al stress, ROS accumulate in plants, resulting in lipid peroxidation in the plasma membrane and production of dysfunctional organelles. Plants enhance enzymatic and nonenzymatic ROS-scavenging systems, such as SOD, POD, catalase (CAT), and ascorbic peroxidase (APX), to scavenge ROS and free radicals under Al stress [56–59]. Previous studies have identified Al-tolerant regions with high antioxidant enzyme activity in the root system of peas, which can mitigate Al toxicity and restore root growth [60–62]. In the present study, POD activity, MDA and H_2_O_2_ contents, and SOD activity were significantly higher in the roots of the *osalr3* mutant and WT lines under Al stress compared with that under normal conditions. Notably, the *osalr3* mutant lines had significantly higher MDA and H_2_O_2_ contents and POD activity and significantly lower SOD activity than the WT plants (Fig. 3), which was consistent with previous findings [63–66]. The damage of Al stress on rice plant is related to oxidative damage. Lipid peroxidation is a classic symptom of oxidative stress. MDA is a product of lipid peroxidation, and its content can reflect the degree of oxidative stress in plants. Al ions readily adhere to the carboxylic and phosphate groups of plasma membranes that can result in lipid peroxidation and the production of MDA as one of the end products of lipid peroxidation [67]. Al treatment increases H_2_O_2_, methylglyoxal (MG), and O_2_^−^, which are related to an increase in plasma membrane peroxidation (MDA content). Overall, Al stress causes a decrease in rice growth and biomass by inducing oxidative stress and biofilm damage [68]. SOD is involved in catalyzing ROS, and SOD activity showed an increase in soybeans, maize, and barley under Al stress [69–71]. SOD may be involved in ameliorating Al-induced oxidative stress by activating antioxidant enzymes associated with the H_2_O_2_ scavenging [72]. Peroxidase plays a vital role in eliminating H_2_O_2_ and harmful substances such as phenols and aldehydes [73]. Compared with that in the WT plant, the expression levels of 30 genes associated with redox processes, including *FeSOD*, *SODcc1*, and *SODcc2*, were significantly downregulated in the *osalr3* mutant lines under Al stress (Fig. 5A). Additionally, *APX1*, *APX8*, *POD2*, *OsPOX8.1*, and *POD22.3*, were significantly higher in the WT (Fig. 5B). Collectively, these results indicate that the *osalr3* mutants were more susceptible to oxidative stress than the WT plant, as evidenced by the low expression of ROS scavenging enzymes, further confirming the involvement of *OsAlR3* in Al stress tolerance in rice.

Taylor [74] proposed two potential mechanisms through which plants respond to Al stress: external detoxification and internal tolerance. The mechanism of external detoxification is primarily located in the plastid bodies of cells. Plant root cells isolate Al ions outside the plastid body, preventing their entry into the interior of the cell. The internal tolerance mechanism occurs at specialized sites within the cytoplasm of the cell. Organic acids with the ability to chelate Al play a crucial role in external and internal detoxification of Al [75]. The amount of organic acids secreted during Al stress varies among plant species. Rye typically secretes more organic acid anions than wheat, whereas rice secretes only a small amount of citrate, which aids in mitigating Al toxicity [76]. Overexpression of genes encoding organic acid synthesis can increase citrate secretion and enhance Al tolerance in crops [19]. For instance, *TAALMT1* overexpression in barley, wheat, and Arabidopsis increases malic acid secretion and Al tolerance [20, 77]. Under Al stress, the secretion of CA decreased in *osalr3* mutant lines (Fig. 3C). Among 21 organic acids detected in rice under Al stress, 12 (including lactic acid and d-lactic acid) were significantly lower in the *osalr3* lines than in the WT plants under Al stress. Additionally, among 13 organic acids detected under normal conditions, only three were significantly lower in *osalr3* lines than in the WT plants (Fig. 6E–F). Overall, these results suggest that the roots of WT plants had a greater ability to secrete Al-chelating organic acids to alleviate Al toxicity. A comprehensive analysis of Al content and organic acid profile of *osalr3* and WT plants showed that Al uptake content was significantly higher in the roots of *osalr3* plants than that in WT plants under Al stress, which was consistent with the decrease in organic acid secretion. Overall, the high concentration of Al in the *osalr3* mutant lines could be mainly attributed to the low secretion of Al-chelating organic acids. Previous studies have shown that the use of organic acids can reduce Al toxicity by increasing plant growth and root activity and reducing lipid peroxidation [78]. Notably, treated with CA and OA restored normal root growth and alleviated Al-induced plasma membrane damage and oxidative stress (Fig. 8). These results indicate that *OsAlR3* knockout decreases organic acids secretion and that organic acid treatment ameliorates Al toxicity.

Phenylpropanoid metabolite content is closely associated with the removal of ROS under both abiotic and biotic stress conditions [79]. The phenylpropanoid biosynthesis pathway is closely related to Al tolerance [37]. In the present study, integrated metabolome and transcriptome analysis revealed significant enrichment of the phenylpropanoid biosynthesis pathway. Compared with that in WT plants, the expression of the peroxidase gene in the phenylpropanoid biosynthesis pathway was significantly downregulated in the *osalr3* mutant lines under Al stress (Fig. 7C). Peroxidases are a class of enzymes found throughout the plant body. Research evidence indicates a close relationship between peroxidases and biotic and abiotic stressors [80, 81]. Al toxicity alters the expression of genes encoding peroxidase, glutathione-S-transferase, and reticulin in Arabidopsis and wheat [82, 83]. Members of this enzyme family are grouped into three classes: Class I, II, and III, among which Class III (E1.11.1.7) peroxidases are specifically found in higher plants [84]. E1.11.1.7 is associated with Al stress, as *AtPER64* is one of the upregulated peroxidases in Arabidopsis during Al stress and its increased expression is linked to secondary cell wall formation in the xylem [85, 86]. Additionally, the *AtPrx64* gene, which encodes a class III plant peroxidase, was upregulated in Arabidopsis during Al stress, and the overexpression of this gene enhanced root growth decreased ROS accumulation in the root system [87]. Overall, the changes observed in rice in the present study may be attributed to *OsAlR3* knockout, suggesting its important role in the phenylpropanoid biosynthesis pathway in response to Al toxicity.

## Conclusion

**Fig. 9:**
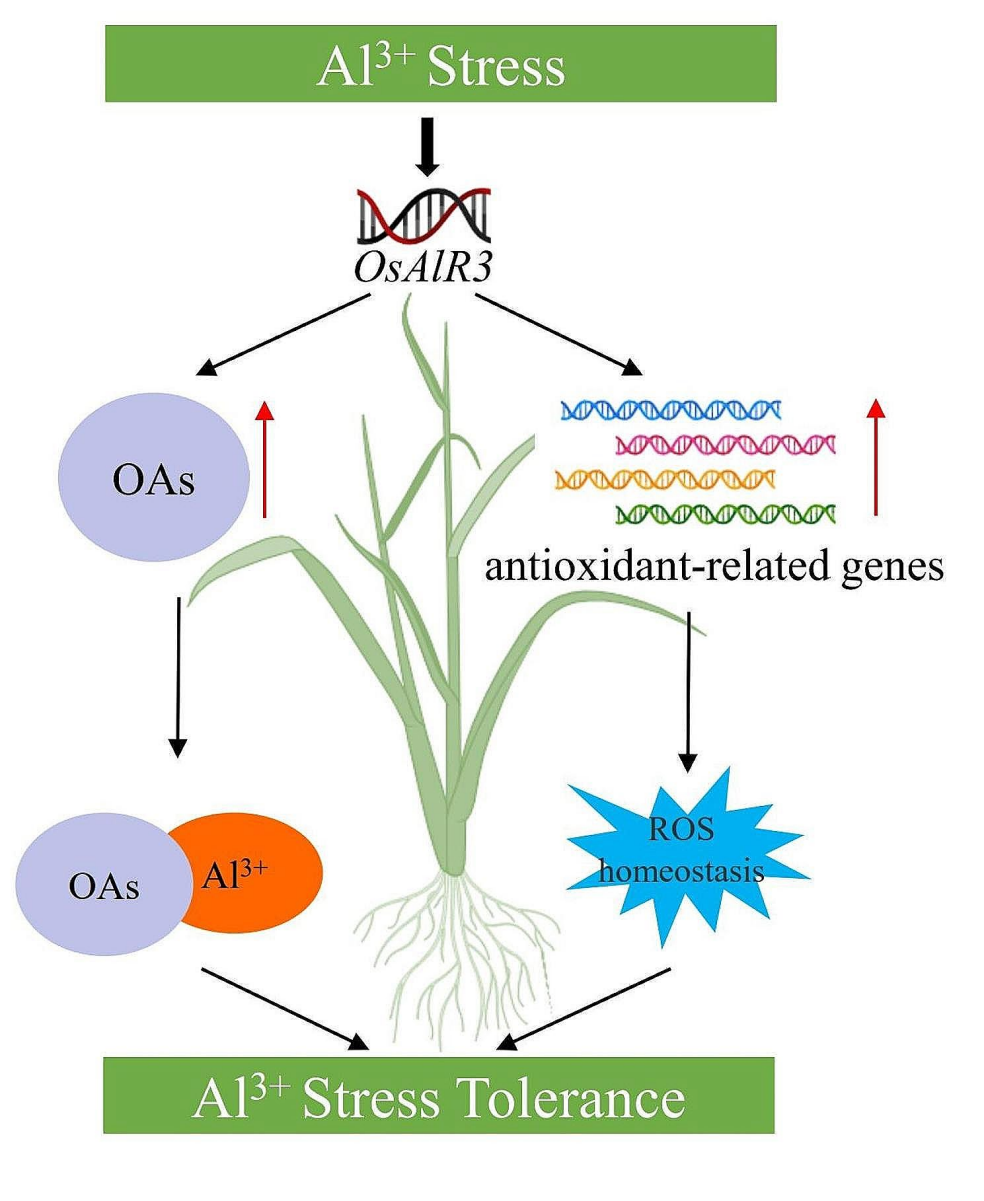
Proposed model of *OsAlR3*-mediated Al tolerance. Al positively regulates the expression of *OsAlR3*. Under Al stress, *OsAlR3* promoted rice roots to secrete organic acids, which chelated Al^3+^ to form non-toxic complexes, and enhanced the resistance of rice roots to Al toxicity. At the same time, *OsAlR3* upregulated the expression of antioxidant-related genes to maintain ROS homeostasis

*OsAlR3* positively regulates Al tolerance in rice. In summary, we propose a model for the role of *OsAlR3* in the response to Al stress in rice (Fig. 9). *OsAlR3* positively regulated Al tolerance and adaptability in rice by increasing the secretion of Al-chelating organic acids and upregulating antioxidant-related genes. *OsAlR3* knockout reduced the secretion of Al-chelating organic acids, increased the concentration of Al^3+^ in the roots, and inhibited root growth. Collectively, these results suggest that *OsAlR3* plays an important role in Al tolerance in rice.

### Electronic supplementary material

Below is the link to the electronic supplementary material.

Supplementary Material 1

Supplementary Material 2

Supplementary Material 3

Supplementary Material 4

Supplementary Material 5

Supplementary Material 6

Supplementary Material 7

Supplementary Material 8

Supplementary Material 9

Supplementary Material 10

Supplementary Material 11

Supplementary Material 12

Supplementary Material 13

Supplementary Material 14

Supplementary Material 15

Supplementary Material 16

Supplementary Material 17

## Data Availability

The datasets presented in this study can be found in online repositories. The names of the repository/repositories and accession number(s) can be found below: https://ngdc.cncb.ac.cn/gsa/, CRA014452.

## Abbreviations

A: adenine deoxyribonucleotide
AAO: ascorbic acid oxidase
Al: aluminum
APX: ascorbic Peroxidase
CA: citric acid
cDNA: complementary DNA
CDS: coding sequence
D: aspartate
DAM: differentially accumulated metabolite
DEG: differentially expressed gene
E1.11.1.7: peroxidase
E2.1.1.104: caffeoyl-CoA O-methyltransferase
FC: fold change
G: glycin
G: guanine deoxyribonucleotide
GO: gene ontology
GWAS: Genome-Wide Association Studies
H2O2: hydrogen peroxide
HCA: hierarchical clustering analysis
HCT: shikimate O-hydroxycinnampyltransferase
KEGG: Kyoto encyclopedia of genes and genomes
MDA: malondialdehyde
MG: methylglyoxal
N: asparagine
O_2_^−^: superoxide anion
OA: oxalic acid
OAs: organic acids
PCA: principal component analysis
PCA: principal component analysis
PLS-DA: Partial Least Squares Discrimination Analysis
PME: pectin methyl esterase
POD: peroxidase
QC: quality control
QTL: Quantitative trait loci
R2: R-squared
REF1: coniferyl-aldehyde dehydrogenase
ROS: reactive oxygen species
RRE: relative root length elongation
RT-qPCR: Real-time Quantitative PCR
sgRNA: single guide RNA
SNP: single nucleotide polymorphism
SOD: superoxide dismutase
T: thymine deoxyribonucleotide
V: valine
VIP: variable importance in projection
WT: wild type
