# Supplementary material for: OsAlR3 regulates aluminum tolerance through promoting the secretion of organic acids and the expression of antioxidant genes in rice
Source: BMC Plant Biol. 2024 Jun 28;24:618. doi: 10.1186/s12870-024-05298-9 (PMC11212236; doi:10.1186/s12870-024-05298-9)
Supplement: Supplementary file 13 — Supplementary Material 13 [file 12870_2024_5298_MOESM13_ESM.pdf]

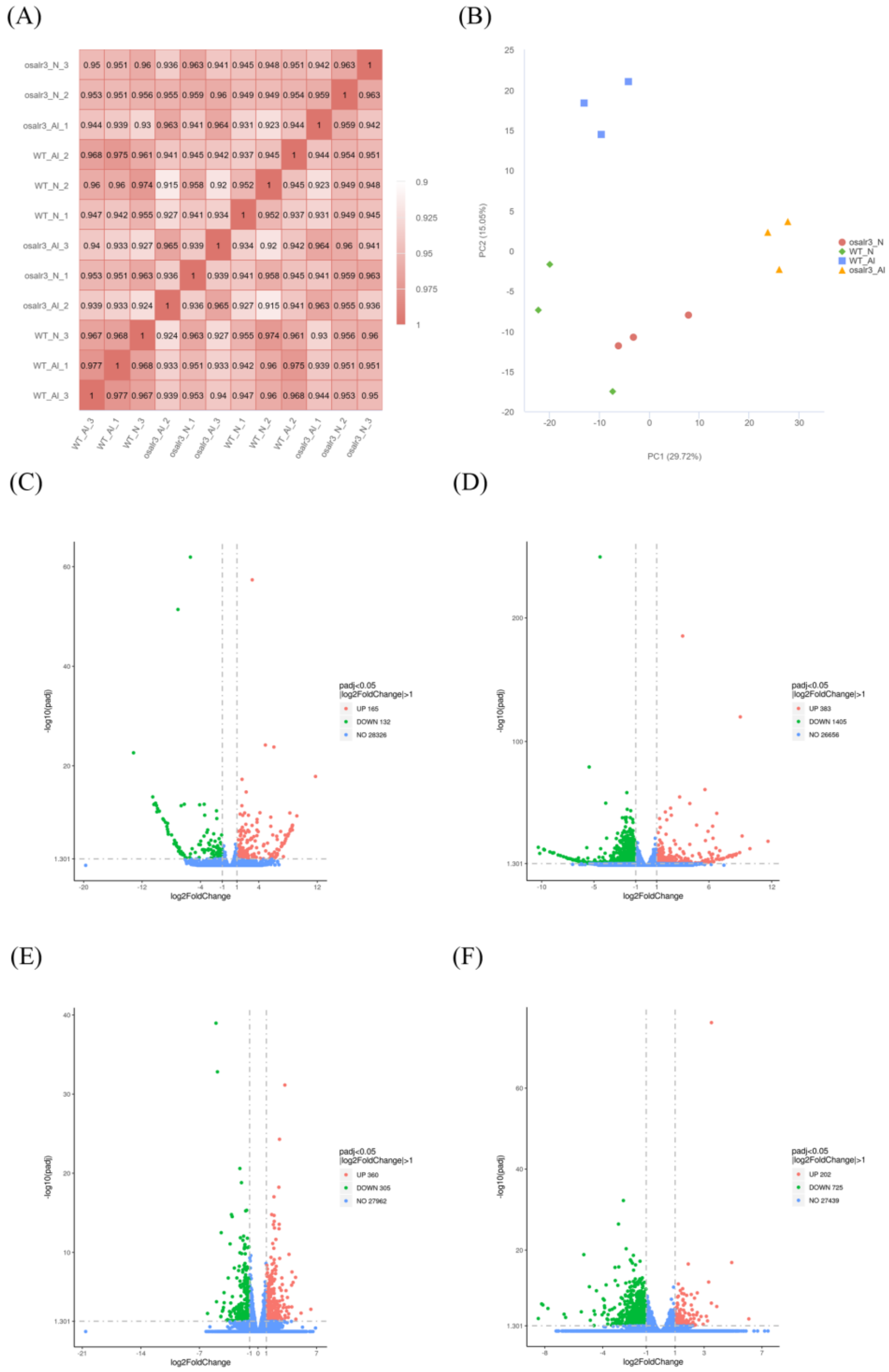

Fig. S1. Overview of the differentially expressed genes (DEGs) between WT and the *osalr3* lines under normal conditions and Al stress. (A) Person correlation coefficient matrix between RNA-Seq samples. (B) PCA analysis of various treatment groups. (C) Volcano plots of the DEGs from the comparison of *osalr3*-N vs. WT-N. (D) Volcano plots of the DEGs from the comparison of *osalr3*-Al vs. WT-Al. (E) Volcano plots of the DEGs from the comparison of WT-Al vs. WT-N. (F) Volcano plots of the DEGs from the comparison of *osalr3*-Al vs. *osalr3*-N. WT-N, WT under normal conditions. WT-Al, WT under Al stress. *osalr3*-N, *osalr3* under normal conditions. *osalr3*-Al, *osalr3* under Al stress.
