# Supplementary figures and images for: OsAlR3 regulates aluminum tolerance through promoting the secretion of organic acids and the expression of antioxidant genes in rice
Source: BMC Plant Biol. 2024 Jun 28;24:618. doi: 10.1186/s12870-024-05298-9 (PMC11212236; doi:10.1186/s12870-024-05298-9)

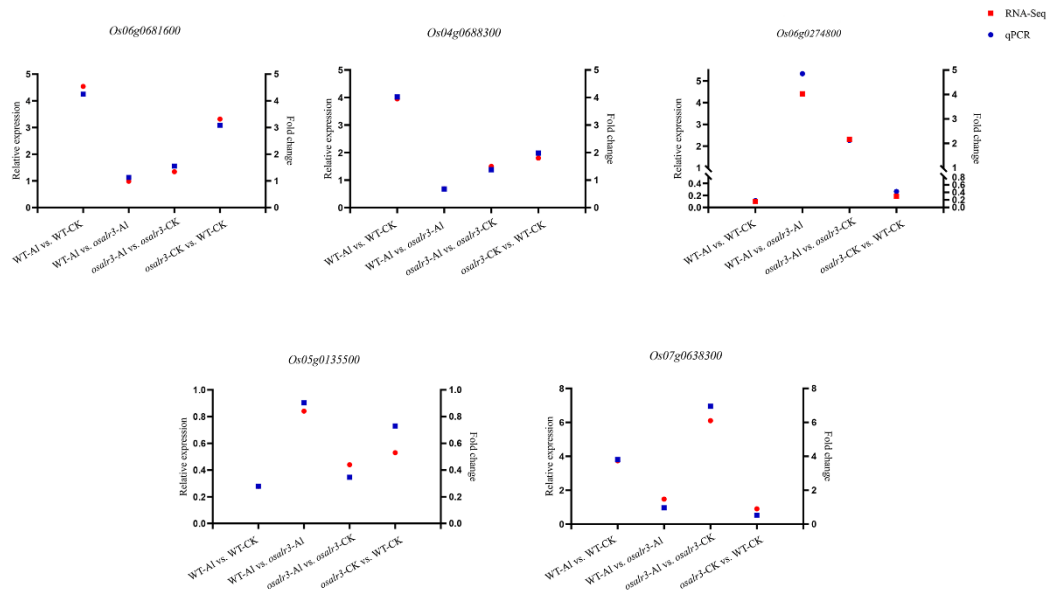

**Fig. S2.** RT-qPCR confirmed the results of RNA-seq analysis.

Supplement: Supplementary file 14 — Supplementary Material 14 [file 12870_2024_5298_MOESM14_ESM.pdf]

(A)

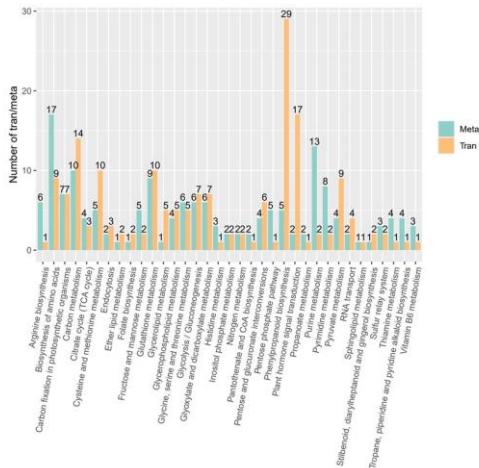

(B)

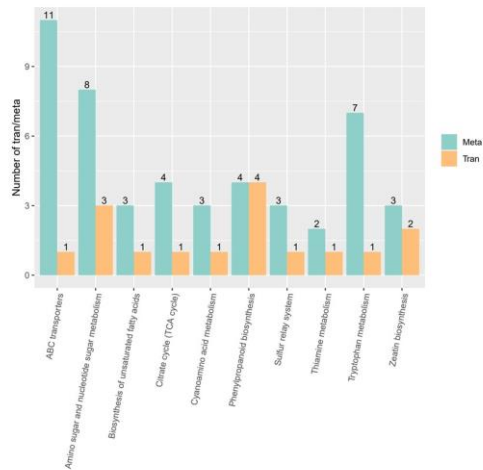

**Fig. S5.** KEGG pathway enrichment analysis of DAMs and DEGs in (A) *osalr3*-Al vs. WT-Al and (B) *osalr3*-N vs. WT-N.

Supplement: Supplementary file 17 — Supplementary Material 17 [file 12870_2024_5298_MOESM17_ESM.pdf]
