# Supplementary material for: OsAlR3 regulates aluminum tolerance through promoting the secretion of organic acids and the expression of antioxidant genes in rice
Source: BMC Plant Biol. 2024 Jun 28;24:618. doi: 10.1186/s12870-024-05298-9 (PMC11212236; doi:10.1186/s12870-024-05298-9)
Supplement: Supplementary file 15 — Supplementary Material 15 [file 12870_2024_5298_MOESM15_ESM.pdf]

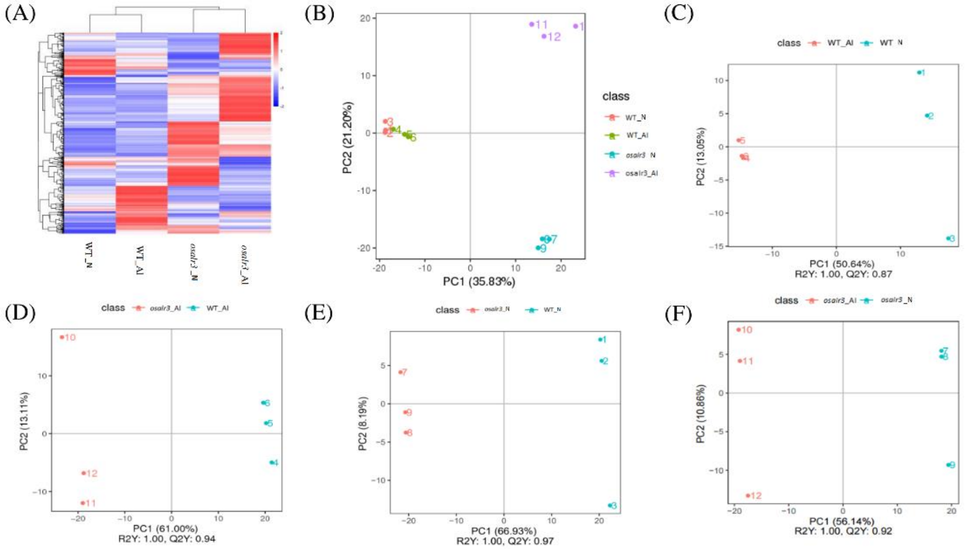

Fig. S3. Overview of the metabolite accumulation under normal conditions and Al stress. (A) HCA of total metabolites in all samples. (B) PCA plots of WT-Al, WT, *osalr3*-Al, *osalr3*-N and QC. (C-F) Sore scatter plots of PLS-DA model for WT-Al vs. WT-N, *osalr3*-Al vs. WT-Al, *osalr3*-Al vs. *osalr3*-N, and *osalr3*-N vs. WT-N.
