# Supplementary material for: OsAlR3 regulates aluminum tolerance through promoting the secretion of organic acids and the expression of antioxidant genes in rice
Source: BMC Plant Biol. 2024 Jun 28;24:618. doi: 10.1186/s12870-024-05298-9 (PMC11212236; doi:10.1186/s12870-024-05298-9)
Supplement: Supplementary file 16 — Supplementary Material 16 [file 12870_2024_5298_MOESM16_ESM.pdf]

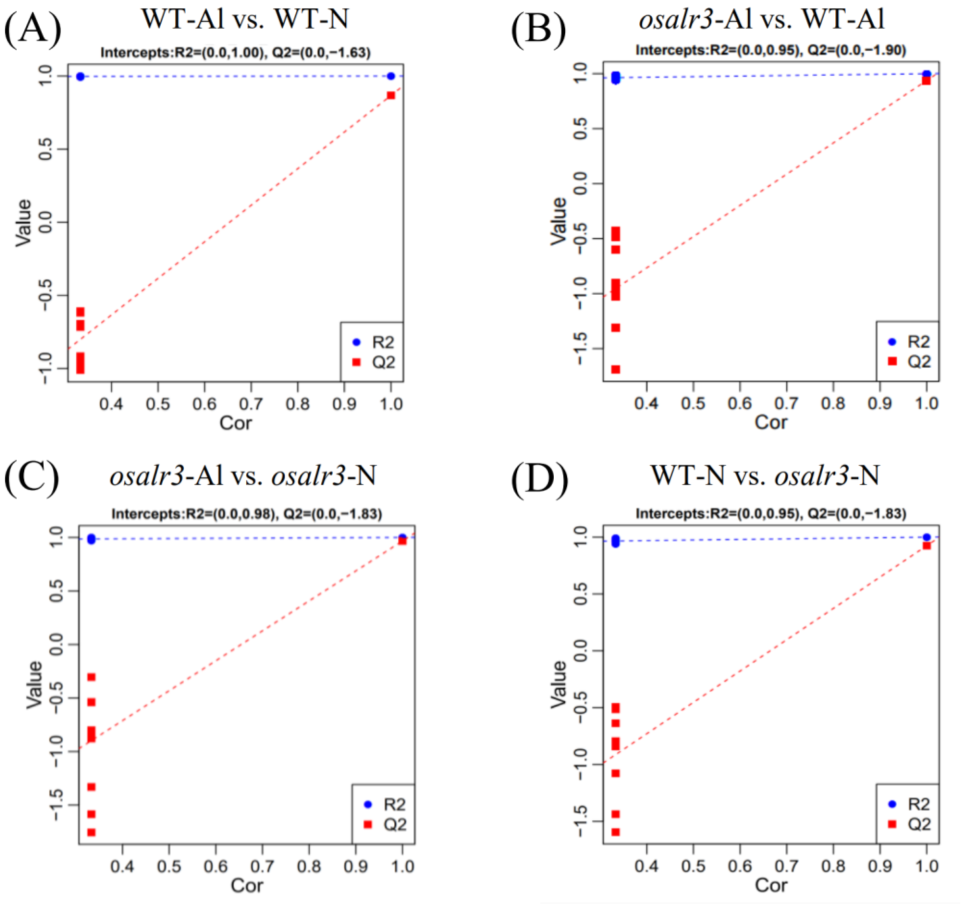

Fig. S4. (A-D) Valid test of PLS-DA model for WT-Al vs. WT-N, *osalr3*-Al vs. WT-Al, *osalr3*-Al vs. *osalr3*-N and WT-N vs. *osalr3*-N.
